# Anisakiasis mimics cancer recurrence: two cases of extragastrointestinal anisakiasis suspected to be recurrence of gynecological cancer on PET-CT and molecular biological investigation

**DOI:** 10.1186/s12880-016-0134-z

**Published:** 2016-04-26

**Authors:** Yuya Nogami, Yoko Fujii-Nishimura, Kouji Banno, Atsushi Suzuki, Nobuyuki Susumu, Taizo Hibi, Koji Murakami, Taketo Yamada, Hiromu Sugiyama, Yasuyuki Morishima, Daisuke Aoki

**Affiliations:** Department of Obstetrics and Gynecology, Keio University School of Medicine, Shinanomachi 35 Shinjuku-ku, Tokyo, 160-8582 Japan; Department of Pathology, Keio University School of Medicine, Shinanomachi 35 Shinjuku-ku, Tokyo, 160-8582 Japan; Department of Surgery, Keio University School of Medicine, Shinanomachi 35 Shinjuku-ku, Tokyo, 160-8582 Japan; Department of Radiology, Keio University School of Medicine, Shinanomachi 35 Shinjuku-ku, Tokyo, 160-8582 Japan; Division of Diagnostic Pathology, Keio University School of Medicine, Shinanomachi 35 Shinjuku-ku, Tokyo, 160-8582 Japan; Department of Parasitology, National Institute of Infectious Diseases, Toyama 1-23-1 Shinjuku-ku, Tokyo, 162-8640 Japan; Department of Pathology, Saitama Medical University, Moroyama-machi 38, Iruma-gun, Saitama, 350-0495 Japan

**Keywords:** Extragastrointestinal anisakiasis, *Anisakis*, PET-CT, Gynecological cancer, Genetic examination, PCR, Glucose transporter type 1, Hexokinase type 2

## Abstract

**Background:**

We report two cases of anisakiasis lesions that were initially suspected to be recurrence of gynecological cancer by positron emission tomography-computed tomography (PET-CT). Both cases were extragastrointestinal anisakiasis that is very rare.

**Case presentation:**

The first case was a patient with endometrial cancer. At 19 months after surgery, a new low density area of 2 cm in diameter in liver segment 4 was found on follow-up CT. In PET-CT, the lesion had abnormal ^18^fluoro-deoxyglucose (FDG) uptake with elevation in the delayed phase, with no other site showing FDG uptake. Partial liver resection was performed. A pathological examination revealed no evidence of malignancy, but showed necrotic granuloma with severe eosinophil infiltration and an irregular material with a lumen structure in the center. Parasitosis was suspected and consultation with the National Institute of Infectious Diseases (NIID) showed the larvae to be *Anisakis simplex* sensu stricto by genetic examination.

The second case was a patient with low-grade endometrial stromal sarcoma (LG-ESS). At 8 months after surgery, swelling of the mediastinal lymph nodes was detected on CT and peripheral T-cell lymphoma was diagnosed by biopsy. A new peritoneal lesion with abnormal FDG uptake was detected on pre-treatment PET-CT and this lesion was increased in size on post-treatment PET-CT. Tumorectomy was performed based on suspected dissemination of LG-ESS recurrence. The findings in a pathological examination were similar to the first case and we again consulted the NIID. The larvae was identified as *Anisakis pegreffi*, which is a rare pathogen in humans.

Having experienced these rare cases, we investigated the mechanisms of FDG uptake in parasitosis lesions by immunohistochemical staining using antibodies to glucose transporter type 1 (GLUT-1) and hexokinase type 2 (HK-2). While infiltrated eosinophils were negative, macrophages demonstrated positive for both antibodies. Therefore, mechanisms behind FDG uptake may involve macrophages, which is common among various granulomas. This is the first report to investigate parasitosis in such a way.

**Conclusion:**

These cases suggest that anisakiasis is a potential differential diagnosis for a lesion with FDG uptake in PET-CT, and that it is difficult to distinguish this disease from a recurrent tumor using PET-CT alone.

## Background

Positron emission tomography (PET)-computed tomography (CT) is increasingly commonly used for various cancers, especially for detection of a metastasis or recurrent lesion. For gynecological cancers, PET-CT is used for examination of possible malignancy of lesions detected by CT or MRI in postoperative follow-up [1]. A lesion with abnormal uptake indicates possible recurrence, and PET-CT is believed to have better accuracy for determining malignancy compared to CT or MRI [2–4]. Surgery is rarely performed for postoperative recurrence, but an exact diagnosis is difficult without a pathological examination. Thus, a lesion that mimics tumor recurrence can lead to unnecessary surgery, chemotherapy, and radiotherapy.

Anisakiasis is relatively common in East Asia due to consumption of raw fish. Anisakiasis often presents with acute abdominal symptoms caused by an allergic reaction in the gastric mucosa. Anisakisis usually infect gastric or intestinal walls, and the feature of the images including PET-CT have been reported [5, 6]. Extragastrointestinal anisakiasis is less common, but such cases have been reported [7–9]. After penetrating the bowel wall, *Anisakis* produce a granuloma as their bodies collapse with time. Incidental detection of this lesion is difficult to distinguish from recurrence in patients with a history of malignancy. This may result in unnecessary resection, and in some cases the collapsing worm body may make the final diagnosis difficult, even after resection.

We experienced two cases of extragastrointestinal anisakiasis in which a recurrent gynecological tumor was initially suspected on imaging. PET-CT showed that the lesions had abnormal uptake in both cases. Even PET-CT could not distinguish between anisakiasis and a tumor metastasis, and diagnosis in the second case was further complicated by collapse of the worm body. A polymerase chain reaction (PCR) using a specific primer for an *Anisakis*-specific region confirmed anisakiasis in both cases.

Besides, we investigated the mechanism of FDG uptake to granuloma of parasitosis. These cases are rare but reported already, however, the mechanisms are still unknown. We investigated it by immunostaining using anti-bodies to glucose transporter type 1 (GLUT-1) and hexokinase type 2 (HK-2), which are recognized as the key factors of FDG uptake in PET.

We describe these two cases with a literature review and molecular biological investigation. We suggest that anisakiasis should be a differential diagnosis for a solitary lesion detected on PET-CT, and we show the utility of PCR for diagnosis of anisakiasis.

## Case presentation

### Case 1

The patient was a 44-year-old woman who had been diagnosed with endometrial cancer. She underwent semi-radical hysterectomy, bilateral salpingo-oophorectomy, pelvic and paraaortic lymph node dissection, and partial omentectomy. A pathological examination revealed that the tumor was grade 2 endometrioid adenocarcinoma with more than 50 % myometrial invasion, and clinical stage Ic (FIGO 1988). Given the intermediate risk of recurrence based on the pathological result, she received adjuvant chemotherapy of 6 cycles of a cyclophosphamide-adriamycin-cisplatin (CAP) regimen.

**Fig. 1:**
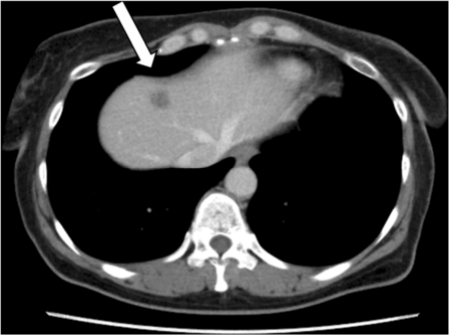
Axial slice on enhanced CT in the equilibrium phase. A low density area was detected in segment 4 of the liver (*arrow*)

**Fig. 2:**
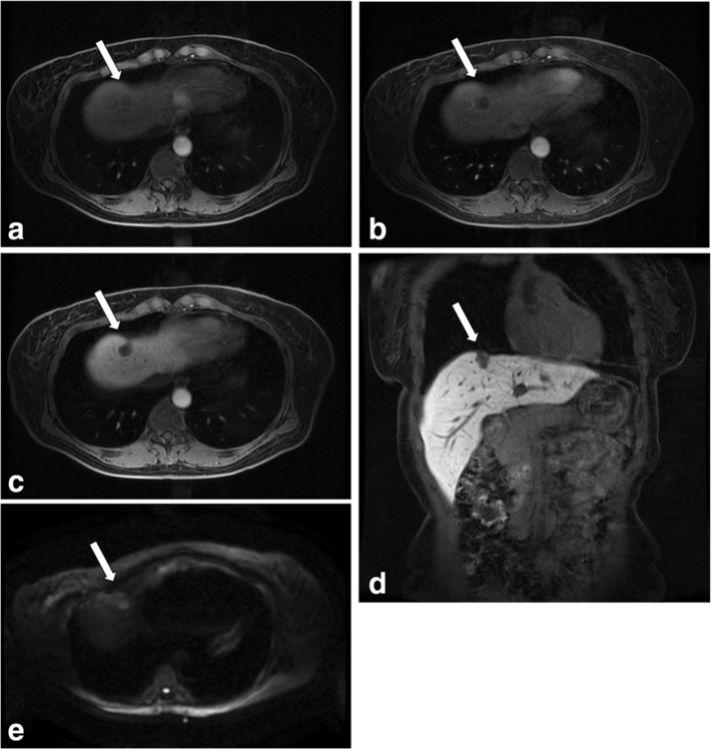
Axial slices of liver acquisition with volume acceleration flex on dynamic MRI in pre-contrast phase (**a**), arterial phase (**b**) and the hepatobiliary phase (**c**). Coronal slice on enhanced MRI in the hepatobiliary phase (**d**). The lesion (*arrows*) gave a low intensity signal in all phases and a high intensity signal in DWI (**e**)

Follow-up CT performed 19 months after surgery detected a new low density area of 15 mm in diameter in segment 4 of the liver (Fig. 1). Serum tumor markers including CA125 and CA19-9 were not elevated, and were similar to the levels before initial therapy. Data from blood tests are shown in Table 1. MRI showed a liver tumor with a high intensity signal in diffusion-weighted imaging (DWI). In a dynamic study using gadolinium ethoxybenzyl diethylenetriaminepentaacetic acid (Gd-EOB-DTPA), early staining in the arterial phase was unclear and the lesion gave a low intensity signal in the hepatobiliary phase (Fig. 2). These findings were compatible with a metastatic tumor.

**Table 1:** Data from blood test of case 1. Eosinophilia was not shown and no tumor marker was elevated

TB	0.8	mg/dl	(0.4–1.2)	WBC	6000	/μl	(3600–8900)	AFP	1	ng/ml	(<20)
AST	17	U/L	(5–37)	Neutro	55.2	%	(37–72)	PIVKA-II	12	mAU/ml	(<40)
ALT	15	U/L	(6–43)	Lymph	37.6	%	(25–48)	CA19-9	<1	U/ml	(<35)
ALP	245	U/L	(110–348)	Mono	3.7	%	(2–12)	CA125	16	U/ml	(<37)
γGTP	13	U/L	(0–75)	Eosino	3.7	%	(1–9)				
BUN	11.6	mg/dl	(9–21)	Baso	2.2	%	(0–2)				
Cr	0.62	mg/dl	(0.5–0.8)	Hb	13.9	g/dl	(11.2–15.2)				
				HCT	42	%	(35.6–45.4)				
				Plt	2.43	10^6^/μl	(1.53 - 3.46)				

*TB* total bilirubin, *AST* aspartate aminotransferase, *ALT* alanine aminotransferase, *ALP* alkaline phosphatase, *gGTP* γ glutamyl transpeptidase, *BUN* blood urea nitrogen, *Cr* creatinine, *WBC* white blood cell, *Neutro* neutrophil, *Lymph* lymphocyte, *Mono* monocyte, *Eosino* eosinophil, *Baso* basophil, *Hb* hemogrobin, *HCT* haematocrit, *Plt* platelet, *AFP* alpha‐fetoprotein, *PIVKA-II* protein induced by vitamin K absence or antagonist-II

**Fig. 3:**
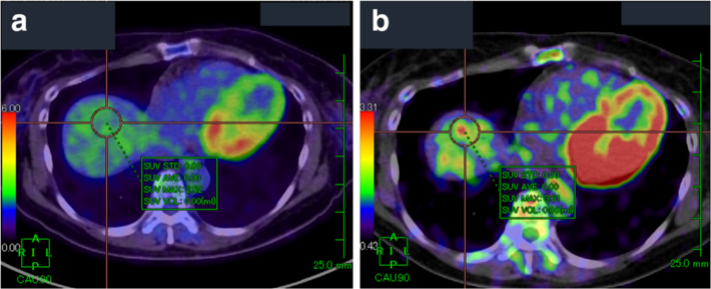
Axial slice on PET-CT showing the liver lesion in the early (**a**) and delayed phase (**b**). FDG uptake was undetectable among physiological uptake of the liver in the early phase; however, uptake elevated (SUVmax: 2.52 to 3.61) and was detectable in the delayed phase

PET-CT was performed to confirm the presence of a malignant liver tumor and to search for other metastases. The liver lesion had no specific ^18^fluoro-deoxyglucose (FDG) uptake compared with normal liver tissue in the early phase, and this was elevated in the delayed phase (standardized uptake value (SUV) max: 2.52 in the early phase, 3.61 in the delayed phase) (Fig. 3). No other metastasis was detected. Recurrence of endometrial cancer was suspected and partial resection of the liver was planned for the solitary metastasis.

**Fig. 4:**
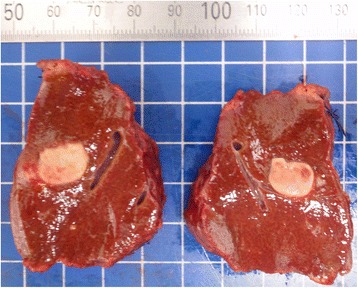
Macroscopic findings in the resected liver, showing a white node with a regular border of about 2 cm in diameter

**Fig. 5:**
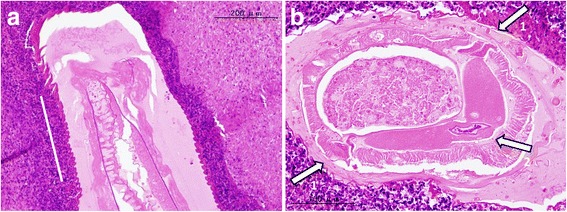
**a** Sagittal slice of the larva, showing a wave-shaped (cross-striated) border (*white line*). **b** Axial slice of the larva, showing a Y-shaped lateral cord (*arrows 1*) and a renette cell (*arrow 2*). These findings are characteristics of *Anisakis*

The resected liver sample included a white nodule of 17 mm in diameter with a regular border macroscopically (Fig. 4). Microscopic examination showed clear eosinophil infiltration and granuloma with an exogenous material in the center. There were no findings consistent with malignancy. The exogenous material had a lumen structure which was suspected to be due to larva migrans. The slide was sent to the National Institute of Infectious Diseases (NIID) to identify the larvae. A detailed microscopic examination revealed that the larvae had Y-shape lateral cords and renette cells, which are specific to *Anisakis* (Fig. 5).

**Fig. 6:**
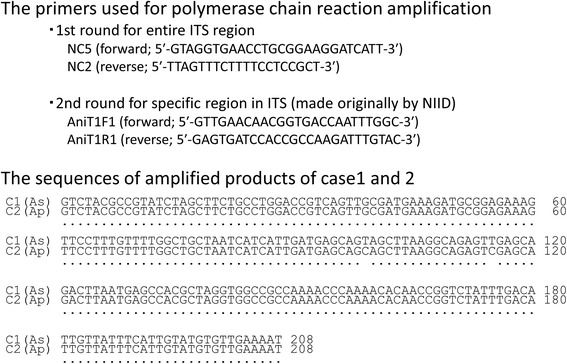
Primers used for genetic identification of *Anisakis* and sequences obtained

A PCR method using a specific primer pair was performed for identification of the *Anisakis* species. The methods of DNA amplification and sequencing are described under a separate heading. This search revealed that the larvae were *Anisakis simplex* sensu stricto (Fig. 6).

These findings showed that the liver lesion was not due to recurrence of endometrial cancer. The patient has had no recurrence for 4 years.

### Case 2

The patient was a 33-year-old woman who had been diagnosed with low-grade endometrial stromal sarcoma (LG-ESS). She underwent extended total hysterectomy and bilateral salpingo-oophorectomy. Pathological examination revealed clinical stage IB (FIGO 2008). High-dose medroxyprogesterone acetate (400 mg/day) was administered as adjuvant therapy.

**Fig. 7:**
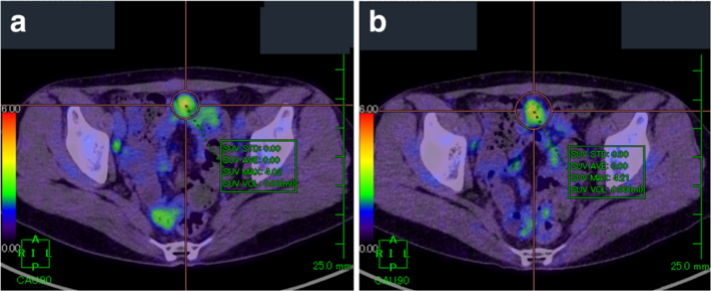
Axial slice on PET-CT after CHOP therapy in the early (**a**) and delayed phase (**b**). The lesion had increased in size compared with pre-treatment and showed FDG uptake (SUVmax: 4.02 to 4.21)

Follow-up CT 8 months after surgery indicated swelling of mediastinal lymph nodes. Biopsy of these nodes performed by a respiratory surgeon revealed peripheral T-cell lymphoma (PTCL), rather than recurrence of LG-ESS. She was referred to the department of hematology. PET-CT performed for pretreatment staging showed abnormal FDG uptake in a nodule of 10 mm in diameter in the peritoneum just below the lower median abdominal wall, in addition to uptakes in mediastinal lymph nodes. The nodule was located clearly extragastrointestinally. The patient was treated with 3 cycles of a cyclophosphamide-adriamycin-vincristin- prednisolone (CHOP) regimen. Post-treatment PET-CT showed that the nodule in the peritoneum increased in size to 16 mm with abnormal FDG uptake (SUVmax: 4.02 in the early phase, 4.21 in the delayed phase) (Fig. 7), despite a marked effect of the therapy on other lesions. Data from blood tests before chemotherapy are shown in Table 2.

**Table 2:** Data from blood test of case 2. Eosinophilia was not shown and sIL2R was elevated due to lymphoma

TB	0.5	mg/dl	(0.4–1.2)	WBC	5400	/μl	(3600–8900)	sIL2R	381	U/ml	(<20)
AST	13	U/L	(5–37)	Neutro	53.3	%	(37–72)	CA125	11	U/ml	(<37)
ALT	7	U/L	(6–43)	Lymph	34.9	%	(25–48)				
ALP	237	U/L	(110–348)	Mono	5.8	%	(2–12)				
LDH	184	U/L	(0–75)	Eosino	5.4	%	(1–9)				
BUN	10.4	mg/dl	(9–21)	Baso	0.6	%	(0–2)				
Cr	0.6	mg/dl	(0.5–0.8)	Hb	13	g/dl	(11.2–15.2)				
				HCT	39.1	%	(35.6–45.4)				
				Plt	2.5	10^6^/μl	(1.53–3.46)				

*TB* total bilirubin, *AST* aspartate aminotransferase, *ALT* alanine aminotransferase, *ALP*, alkaline phosphatase, *LDH* lactate dehydrogenase, *BUN* blood urea nitrogen, *Cr* creatinine, *WBC* white blood cell, *Neutro* neutrophil, *Lymph* lymphocyte, *Mono* monocyte, *Eosino* eosinophil, *Baso* basophil, *Hb* hemogrobin, *HCT* haematocrit, *Plt* platelet, *sIL-2R* soluble interleukin-2 receptor

**Fig. 8:**
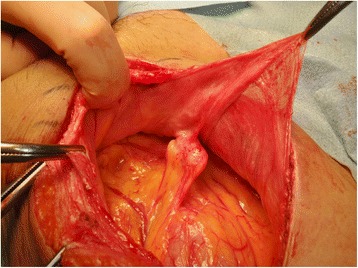
Intra-abdominal findings in exploratory laparotomy. The lesion was located on the omentum with adhesion to the parietal peritoneum

**Fig. 9:**
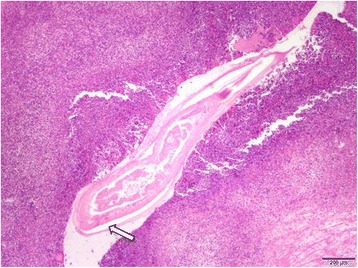
The nodule had an abscess with clear eosinophil infiltration. The larva body had collapsed, but the specific Y-shaped lateral cord was recognizable (arrow)

The PET-CT findings for the nodule were not compatible with PTCL; therefore, exploratory laparotomy was performed to examine possible dissemination of LG-ESS. The nodule was on the omentum and partial omentectomy was performed (Fig. 8). No other macroscopic lesion in the abdomen was found. Pathological examination revealed that the nodule had an abscess with clear eosinophil infiltration. An exogenous material that appeared to be a larva was found in the center of the abscess. The larva body had collapsed, but the specific Y-shape lateral cord was recognizable (Fig. 9). Based on the experience of the first case, we sent the slide to the NIID. The PCR method revealed that the worm was *Anisakis pegreffi* (Fig. 6), which is rare as an infectious pathogen in human. These findings showed that the patient did not have recurrent LG-ESS. The patient has had no recurrence of LG-ESS for 2 years and that of PTCL for a year.

### Imaging parameters of CT, MRI, and PET-CT

CT: The device is an Acquilion ONE (Toshiba Medical Systems, Otawara, Japan) by using a tube voltage of 120 kVp and tube current varied between 96 mA and 160 mA. Slice thickness is 5 mm for abdominal scanning as a post-operative follow up in a cancer patient.

MRI: The device is a Discovery MR750 3.0 T (GEHealthcare, Waukesha, Wisconsin, USA) using 32-channel torso coil as a receiver. Liver acquisition with volume acceleration flex (LAVA-Flex) is practiced with EOB dynamic MRI in our institute. The acquisition sequence is LAVA Flex with parameters as follows: Time of repectition (TR)/time of echo (TE) = 3.9/1.7 msec, flip angle = 12 o, slice thickness/gap = 3.6/1.8 mm, matrices = 256 × 256, acquisition time = 19 s. Gd-EOB-DTPA: Primovist® (Bayer Healthcare AG, Berlin, Germany)-enhanced images in the hepatobiliary phase are taken at 15 min after contrast injection.

PET-CT: Biograph mCT (Siemens Medical Solutions, Knoxville, TN, USA). In our institute, patients routinely receive DPS for differentiation. Patients were administered 3.7 MBq/kg of FDG and received routine PET-CT DPS at 1 and 2 h after administration. Data were analyzed on an AZE workstation (AZE Ltd, Tokyo, Japan).

### Molecular investigation

#### Identification of anisakid nematodes (DNA amplification and sequencing)

The worm body was scratched from the deparaffinized slide under stereoscopic microscopy and placed in a plastic tube. DNA was extracted using proteinase K (Qiagen) and SDS (Sigma-Aldrich) [10]. The entire internal transcribed spacer (ITS) region (ITS1, 5.8S rDNA and ITS2) was amplified by PCR using the primers NC5 and NC2 in the first round. Then nested PCR was performed in the second round to amplify a specific region in the ITS1 using the following primers that were originally constructed: AniT1F1: 5′-GTTGAACAACGGTGACCAATTTGGC-3′, and AniT1R1: 5′-. PCR was conducted by the method indicated in reference [10]. An amplification product of 208 bp was obtained. Sequence similarities were determined by a BLAST search of the DNA Data Bank of Japan (DDBJ) (http://www.ddbj.nig.ac.jp/index-e.html). Sequence alignment and comparison was facilitated by the GENETYX-WIN program (ver. 7.0, Software Development Co, Tokyo, Japan).

#### Investigation for the mechanism of FDG uptake (immunochemical staining using anti-bodies to GLUT-1 and HK-2)

Once FDG is uptaken to cells through the GLUT-1 and phosphorylated by HK-2, FDG is unable to pass out of cells unless dephosphorylated by glucose-6-phosphatase (G6P). Because malignant cells have a high ratio of HK-2/G6P, they present an elevation of FDG uptake in delayed phase in dual-phase scanning (DPS) of PET-CT. Since both cases indicated the elevation, recurrence was a stronger consideration among other differential diagnoses.

Cases of parasitosis suspected as malignancy in PET have been previously reported but are very rare [11–13]. There are no reports investigating the mechanisms of FDG uptake in parasitosis granuloma. We researched them to develop ways to differentiate them by investigating overexpression of GLUT-1 and HK-2.

GLUT-1 expression was evaluated immunohistochemically using rabbit polyclonal anti-Glucose Transporter GLUT1 antibody (ab652, Abcam, Cambridge, UK) at a dilution of 1:500. HK-2 expression was evaluated with rabbit polyclonal anti-Hexokinase Type II (AB3279, Chemicon International, Temecula, USA) diluted at 1:500. Immunohistochemical staining was performed using the Leica Bond-Max automatic immunostainer and the Bond Polymer Refine Detection kit (Leica).

**Fig. 10:**
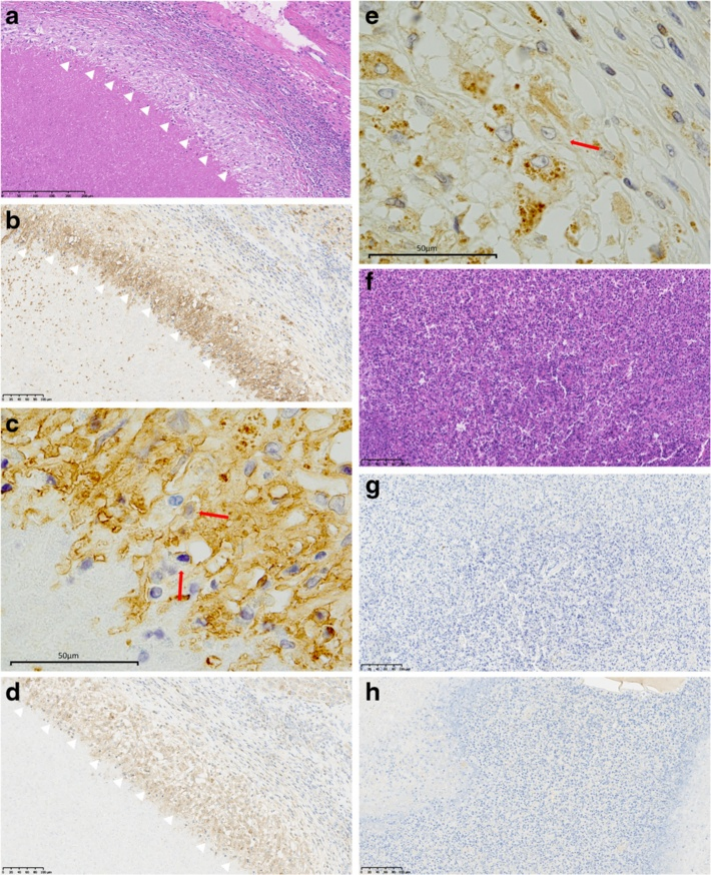
In case 1, macrophages around abscess (**a** HE, *arrows*) were positive for GLUT-1 on the cell membrane (**b**, **c**, *arrows*) and HK-2 in the cytoplasm (**d**, **e**, *arrows*). Extensive eosinophil infiltrate (**f** HE, throughout the whole figure) were negative for GLUT-1 (**g**) and HK-2 (**h**)

**Fig. 11:**
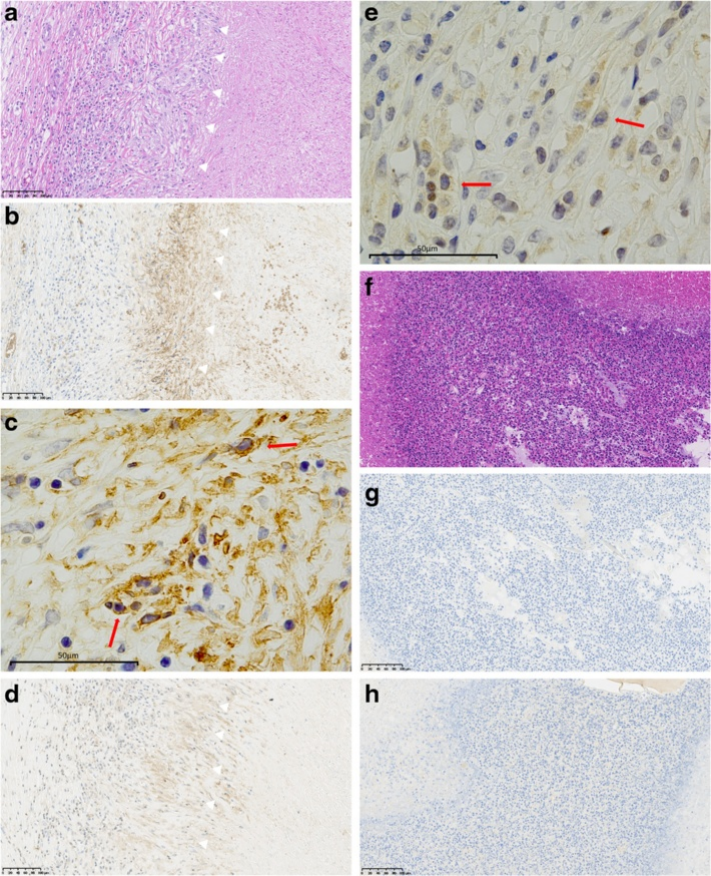
In case 2, macrophages around abscess (**a** HE, *arrows*) were positive for GLUT-1 on the cell membrane (**b**, **c**, *arrows*) and HK-2 in the cytoplasm (**d**, **e**, *arrows*). Extensive eosinophil infiltrate (**f** HE, throughout the whole figure) were negative for GLUT-1 (**g**) and HK-2 (**h**)

In both cases, only macrophages were positive for both antibodies; GLUT-1 and HK-2. The eosinophils, which were infiltrated around worm bodies, were negative for them (Figs. 10, 11). These results suggest that eosinophil infiltration may not boost FDG uptake or have any specific roles. Instead, the reason for FDG uptake to parasitosis granuloma may be the macrophages, which is commonly seen in granulomas.

## Discussion

Extragastrointestinal anisakiasis accounts for 0.45 % of cases of anisakiasis in Japan [14]. The condition is caused by an *Anisakis* worm penetrating the bowel wall and forming an intra-abdominal colony. Cases in the omentum have been described in Japan and Italy [7, 15]. Occurrence as a solitary liver lesion is particularly rare, with the only examples found in eight cases reported in Japan [16, 17]. Most cases of extragastrointestinal anisakiasis are asymptomatic and detected incidentally.

In the present two cases, malignant metastatic tumor was indicated as first choice of differential diagnosis by two imaging modalities. Dynamic MRI using contrast agent is often performed as the detailed examination for hepatic tumor, as in case 1. Low signal in hepatobilliary phase without early enhancement is compatible with metastatic tumor. However, because Gd-EOB-DTPA contrasting is dependent on expression of OATP1B1/3 [18], and parasitic granuloma does not necessarily show expression of OATP1B1/3, Gd-EOB-DTPA may not be useful for differentiating between metastatic tumor and parasitic granuloma. Using Gd-EOB-DTPA contrast benefits in clarifying the tumor location. The diagnostic efficacy of PET-CT for extragastrointestinal anisakiasis has not been examined because of the rarity of the disease. Our cases indicated that lesions of extragastrointestinal anisakiasis present with abnormal FDG uptake and were difficult to distinguish from a malignant tumor. *Anisakis* cannot live in the human body and die immediately, and thus FDG uptake was not directly associated with the worm body, but with inflammatory cells around the body. Dual-phase scanning is a well-known method for distinguishing between inflammation and malignancy [19–22], based on the observation that malignant lesions have elevated FDG uptake in the delayed phase compared with the early phase. However, the lesions in both of our cases showed elevation in delayed phases, especially in case 1. Without previous CT or MRI, the tumor might not have been detected using only the early phase scan. There has been a report of an anisakiasis case without FDG uptake [16] and the uptake decreased with time in our second case, suggesting that FDG uptake in anisakiasis lesions may decrease with time. This may help to distinguish it from malignancy, but it remains difficult to observe without therapy for cases in which malignancy is suspected.

Anisakiasis occurs due to eating of infected raw fish, such as mackerel, and horse mackerel. Consumption of raw fish is common in Asia, including Japan, and Northern Europe [23], and there is a recent trend to expansion of this behavior in the United States [24]. Food and drug administration in the United States and European Food Safety Authority in Europe recommend the frozen stock required for raw fish to prevent parasitosis. Increased use of imaging modalities such as MRI and PET-CT is likely to lead to more incidental findings of asymptomatic lesions [25], and this may lead to an increase in cases similar to those in this report. This is a concern because differential diagnosis from a malignant tumor is difficult for these lesions. The difficulty was provided by time lag. There is a time lag between infection and detection of a lesion, since generating a granuloma or an abscess in anisakiasis takes 2 months to 2 years [26]. The prior imaging to the detection of the tumor was 10 months in case 1, and 2 months in case 2. It is suspected that the patients were infected and granuloma formation occurred during this interval. The hematological or serological acute reaction of infection and the patient’s memory of symptoms or food consumption can be lost due to this time lag. In this situation, a test for eosinophilia, a specific antibody test and history taking, which are important in diagnosis of parasitosis, are less useful. In fact, eosinophilia was not seen in either of our cases and a specific antibody for *Anisakis* was negative in the first case. And we retook the patients’ history retrospectively after surgery, neither of the patients remembered a characteristic dietary history. The difficulty of diagnosing is not only because of the vagueness of memory, but is also related to the existence of asymptomatic cases. Our two cases were also asymptomatic cases and there were no imaging findings on contrast CT. Generally, *Anisakis* larvae bite the intestinal wall, which causes acute severe abdominal pain. The pain is caused mainly by allergic reaction, rather than mechanical stimulus. Even in extragastrointestinal anisakiasis, which larvae penetrate the wall of the gastrointestinal tract, many cases are detected incidentally. There seems to be many asymptomatic cases.

The risk of recurrence of a primary malignancy should also be considered. In our first case, the risk of recurrence of endometrial cancer was intermediate, at about 10 % at 20 months after initial therapy [27]. In the second case, the patient had stage I LG-ESS. The recurrence risk for this cancer is over 30 %, even in stage I, although the period of progression-free LG-ESS is generally longer than 8 months [28]. Therefore, both cases had a relatively low risk of recurrence, but this information was difficult to include in the diagnosis with certainty. In addition to the complexity of FDG uptake in PET-CT, the lesion location may also increase the difficulty of diagnosis. If a lesion is detected in the intestinal wall or intramesentery, in which anisakiasis is relatively common, recurrence of gynecological cancer is less likely. However, a lesion in the peritoneum or liver is less likely to be due to extragastrointestinal anisakiasis, and this is also a common site of tumor recurrence. Cases of extragastrointestinal anisakiasis in the uterus [29, 30] and ovary [31] have also been reported, indicating that differential diagnosis of such lesions is also required.

Generally, inflammatory lesions such as pneumonia and abscess, which need to be differentiated with malignancy, include macrophages and neutrophils. These cells increase the glucose uptake, but the changes seen in the delayed phase in DPS differs with cancer cells because of the difference of HK-2/G6P ratio. The two cases we report had eosinophilic granuloma. We speculated that the elevation of SUV in the delayed phase may be caused by eosinophils, and investigated their feature by immunochemical straining using anti-GLUT-1 and anti-HK-2 antibodies. However, against our expectation, eosinophils showed negative expression of GLUT-1 and HK-2, and only macrophages were strained. Although we have not investigated the expression of G6P, the expression HK-2 in macrophages may cause the elevation of uptake in inflammatory lesion in is the delayed phase. Thus, DPS is not necessarily superior to other modalities in distinguishing malignancy and inflammation. New imaging modalities such as PET-CT allow an “abnormality” to be detected incidentally. It is important to determine whether a lesion found on PET-CT is truly abnormal, and this limitation of new technologies should be recognized.

In our cases, a genetic test was used to define the pathogen. The *Anisakis* worm collapses in the human body and this may cause difficulty with diagnosis, as in our second case. The PCR methods described here are useful in these cases. In addition, improved definition of species among *Anisakis* larvae might bring new insights. No studies have been reported about the frequency of species in extragastrointestinal anisakiasis. However, Umehara A. et al. reported that 99 % of all human anisakiasis are due to *Anisakis simplex* sensu stricto [32]. *Anisakis pegreffi* is relatively common among fish that are landed [33]. This discrepancy is explained by the difference of the larvae’s ability to penetrate to the fish muscle, thus causing ingested by human [34–36]. In both cases included in the present study, it is thought that the larvae penetrated the gastrointestinal wall. Thus, the case of extragastrointestinal anisakiasis of *Anisakis pegreffi* is very rare and interesting. Accumulation of cases are required to clarify the epidemiology of extragastrointestinal anisakiasis and identify routes of infection.

## Conclusion

We experienced two cases of anisakiasis that were initially suspected to be recurrence of gynecological cancer on PET-CT. These cases suggest that anisakiasis should be a differential diagnosis for a lesion presenting with FDG uptake on PET-CT. Our results also indicate that it is difficult to distinguish anisakiasis from a recurrent tumor using PET-CT and there is no specific mechanism of FDG uptake in parasitosis granuloma. These findings are important because current dietary habits and use of imaging modalities suggest that similar cases will increase worldwide.

## Consent for publication

Written informed consent was obtained from the patients for publication of this case series and any accompanying images.

## Availability of data and materials

The dataset supporting the conclusions of this article is included within the article.

## Abbreviations

CT: computed tomography
PET: positron emission tomography
FDG: ^18^fluoro-deoxyglucose
NIID: the National Institute of Infectious Diseases
LG-ESS: low-grade endometrial stromal sarcoma
PCR: polymerase chain reaction
GLUT-1: glucose transporter type 1
HK-2: hexokinase type 2
CAP: cyclophosphamide-adriamycin-cisplatin
FIGO: International federation of gynecology and obstetrics
Gd-EOB-DTPA: gadolinium ethoxybenzyl diethylenetriaminepentaacetic acid
DWI: diffusion-weighted imaging
SUV: standardized uptake value
PTCL: peripheral T-cell lymphoma
CHOP: cyclophosphamide-adriamycin-vincristin- prednisolone
ITS: internal transcribed spacer
DDBJ: DNA Data Bank of Japan
G6P: glucose-6-phosphatase
DPS: dual-phase scanning

## References

[1] Nogami Y, Iida M, Banno K, Kisu I, Adachi M, Nakamura K (2014). Application of FDG-PET in cervical cancer and endometrial cancer: utility and future prospects. Anticancer Res.

[2] Chu Y, Zheng A, Wang F, Lin W, Yang X, Han L (2014). Diagnostic value of 18 F-FDG-PET or PET-CT in recurrent cervical cancer: a systematic review and meta-analysis. Nucl Med Commun.

[3] Gu P, Pan LL, Wu SQ, Sun L, Huang G (2009). CA 125, PET alone, PET-CT, CT and MRI in diagnosing recurrent ovarian carcinoma: a systematic review and meta-analysis. Eur J Radiol.

[4] Antunovic L, Cimitan M, Borsatti E, Baresic T, Sorio R, Giorda G (2012). Revisiting the clinical value of 18 F-FDG PET/CT in detection of recurrent epithelial ovarian carcinomas: correlation with histology, serum CA-125 assay, and conventional radiological modalities. Clin Nucl Med.

[5] Abe K, Yoshikai T, Baba S, Isoda T, Honda H (2014). PET/CT findings in acute gastric anisakiasis. Clin Nucl Med.

[6] Shibata E, Ueda T, Akaike G, Saida Y (2014). CT findings of gastric and intestinal anisakiasis. Abdom Imaging.

[7] Kagei N, Orikasa H, Hori E, Sannomiya A, Yasumura Y (1995). A case of hepatic anisakiasis with a literal survey for extra-gastrointestinal anisakiasis. Jpn J Parasitol.

[8] Cancrini G, Magro G, Giannone G (1997). [1st case of extra-gastrointestinal anisakiasis in a human diagnosed in Italy]. Parassitologia.

[9] Takekawa Y, Kimura M, Sakakibara M, Yoshii R, Yamashita Y, Kubo A (2004). Two cases of parasitic granuloma found incidentally in surgical specimens. Jpn J Clin Pathol.

[10] Sugiyama H, Singh TS, Rangsiruji A, Liu D (2013). Paragonimus. Molecular Detection of Human Parasitic Pathogens.

[11] Yoo Ie R, Park HJ, Hyun J, Chung YA, Sohn HS, Chung SK (2006). Two cases of pulmonary paragonimiasis on FDG-PET CT imaging. Ann Nucl Med.

[12] Chen CJ, Chou SC, Chen HJ, Chen HY (2010). Solitary necrotic nodule with larval infestation in the liver on F-18 FDG PET/CT. Clin Nucl Med.

[13] Cheng W, Li F, Zhuang H, Zhong D, Wu C, Zhu Z (2010). Hepatic paragonimiasis revealed by FDG PET/CT. Clin Nucl Med.

[14] Ishikura H, Kikuchi H, Sato N, Ohtani S, Yagi K, Ishikura H (1992). [Changing larva migrans caused by anisakidae larvae]. Clin Parasitol.

[15] Pampiglione S, Rivasi F, Criscuolo M, De Benedittis A, Gentile A, Russo S (2002). Human anisakiasis in Italy: a report of eleven new cases. Pathol Res Pract.

[16] Ishida M, Sano S, Terada T, Mitsui T, Sudoh Y, Yamaguchi A (2013). A case of hepatic anisakiasis. J Jpn Surg Assoc.

[17] Morita M, Soyama A, Takatuki M, Kuroki T, Abe K, Hayashi T (2013). A case of hepatic mass induced by extra-gastrointestinal anisakiasis. J Jpn Surg Assoc.

[18] Leonhardt M, Keiser M, Oswald S, Kuhn J, Jia J, Grube M (2010). Hepatic uptake of the magnetic resonance imaging contrast agent Gd-EOB-DTPA: role of human organic anion transporters. Drug Metab Dispos.

[19] Zhuang H, Pourdehnad M, Lambright ES, Yamamoto AJ, Lanuti M, Li P (2001). Dual time point 18 F-FDG PET imaging for differentiating malignant from inflammatory processes. J Nucl Med.

[20] Shen G, Hu S, Deng H, Jia Z (2014). Diagnostic value of dual time-point 18 F-FDG PET/CT versus single time-point imaging for detection of mediastinal nodal metastasis in non-small cell lung cancer patients: a meta-analysis. Acta Radiol.

[21] Mochizuki Y, Omura K, Nakamura S, Harada H, Shibuya H, Kurabayashi T (2012). Preoperative predictive model of cervical lymph node metastasis combining fluorine-18 fluorodeoxyglucose positron-emission tomography/computerized tomography findings and clinical factors in patients with oral or oropharyngeal squamous cell carcinoma. Oral Surg Oral Med Oral Pathol Oral Radiol.

[22] Nogami Y, Banno K, Irie H, Iida M, Masugi Y, Murakami K (2015). Efficacy of 18-FDG PET-CT Dual-phase Scanning for Detection of Lymph Node Metastasis in Gynecological Cancer. Anticancer Res.

[23] EFSA Panel on Biological Hazards (2011). Scientific Opinion on assessment of epidemiological data in relation to the health risks resulting from the presence of parasites in wild caught fish from fishing grounds in the Baltic Sea. EFSA Journal.

[24] Fein SB, Lando AM, Levy AS, Teisl MF, Noblet C (2011). Trends in U.S. consumers’ safe handling and consumption of food and their risk perceptions, 1988 through 2010. J Food Prot.

[25] Organisation for Economic Co-operation and Development, OECD Health Statistics 2014. 2014. http://www.oecd.org/health. Accessed 20 May 2015.

[26] Kojima K, Koyanagi T, Shiraki K (1966). Pathological analysis of anisakiasis. Jpn J Clin Med.

[27] Susumu N, Sagae S, Udagawa Y, Niwa K, Kuramoto H, Satoh S (2008). Randomized phase III trial of pelvic radiotherapy versus cisplatin-based combined chemotherapy in patients with intermediate- and high-risk endometrial cancer: a Japanese Gynecologic Oncology Group study. Gynecol Oncol.

[28] Li AJ, Giuntoli RL, Drake R, Byun SY, Rojas F, Barbuto D (2005). Ovarian preservation in stage I low-grade endometrial stromal sarcomas. Obstet Gynecol.

[29] Takao Y, Fukuma T, Shigeki M, Shyono Y, Tokunaga K, Uchiyama A (1993). [An Anisakis larva observed in the utero-cervix]. Clin Parasitol.

[30] Shiozaki Y, Kudo M, Adachi T, Takizawa K, Iguchi T, Takeda Y (1993). A case of anisakiasis by the histological examination after the operation of uterus. Clin Parasitol.

[31] Mori H, Hirata M, Kase Y, Takagi Y, Sekine I, Aoki Y (1982). A case of ovarian anisakiasis. Sankatofujinka.

[32] Umehara A, Kawakami Y, Araki J, Uchida A (2007). Molecular identification of the etiological agent of the human anisakiasis in Japan. Parasitol Int.

[33] Umehara A, Kawakami Y, Ooi HK, Uchida A, Ohmae H, Sugiyama H (2010). Molecular identification of Anisakis type I larvae isolated from hairtail fish off the coasts of Taiwan and Japan. Int J Food Microbiol.

[34] Suzuki J, Murata R, Hosaka M, Araki J (2010). Risk factors for human Anisakis infection and association between the geographic origins of Scomber japonicus and anisakid nematodes. Int J Food Microbiol.

[35] Arizono N, Yamada M, Tegoshi T, Yoshikawa M (2012). Anisakis simplex sensu stricto and Anisakis pegreffii: biological characteristics and pathogenetic potential in human anisakiasis. Foodborne Pathog Dis.

[36] del Carmen RM, Valero A, Navarro-Moll MC, Martin-Sanchez J (2013). Experimental comparison of pathogenic potential of two sibling species Anisakis simplex s.s. and Anisakis pegreffii in Wistar rat. Trop Med Int Health.

